# *Musa* Germplasm A and B Genomic Composition Differentially Affects Their Susceptibility to Banana Bunchy Top Virus and Its Aphid Vector, *Pentalonia nigronervosa*

**DOI:** 10.3390/plants11091206

**Published:** 2022-04-29

**Authors:** Sergine Ngatat, Rachid Hanna, Jules Lienou, Richard T. Ghogomu, Sidonie Prisca K. Nguidang, Aime C. Enoh, Bertrand Ndemba, Sam Korie, Apollin Fotso Kuate, Samuel Nanga Nanga, Komi K. M. Fiaboe, P. Lava Kumar

**Affiliations:** 1 International Institute of Tropical Agriculture (IITA), Messa-Yaoundé P.O. Box 2008, Cameroon; rachidhanna01@gmail.com (R.H.); j.lienou@cgiar.org (J.L.); koumousidonie@yahoo.fr (S.P.K.N.); a.enoh@cgiar.org (A.C.E.); a.fotso@cgiar.org (A.F.K.); s.nanga@cgiar.org (S.N.N.); k.fiaboe@cgiar.org (K.K.M.F.); 2 Department of Plant Protection, University of Dschang, Dschang P.O. Box 96, Cameroon; rt.ghogomu@yahoo.com; 3 Center for Tropical Research, Institute of the Environment and Sustainability, University of California, Los Angeles, CA 90095, USA; 4 Departments of Microbiology and Parasitology, University of Buea, Buea P.O. Box 63, Cameroon; 5 National Program of Fruit Crops (NPFC), Ministry of Agriculture and Rural Development (MINADER), Yaoundé P.O. Box 57, Cameroon; bertrandndemba@yahoo.fr; 6 IITA, PMB 5320 Oyo Road, Ibadan 200285, Nigeria; s.korie@cgiar.org (S.K.); l.kumar@cgiar.org (P.L.K.)

**Keywords:** banana, plantain, A and B genome, host plant resistance, virus transmission, aphid vector, Africa

## Abstract

Banana bunchy top disease (BBTD), caused by the banana bunchy top virus (BBTV, genus *Babuvirus*), is the most destructive viral disease of banana and plantain (*Musa* spp.). The virus is transmitted persistently by the banana aphid, *Pentalonia nigronervosa* Coquerel (Hemiptera: Aphididae). While research efforts have focused on screening *Musa* genotypes for BBTD resistance, comparatively little work has been carried out to identify resistance to banana aphids. This study assessed 44 *Musa* germplasm of different A and B genome composition for the performance of banana aphids under semicontrolled environmental screenhouse conditions and in a field trial established in a BBTD endemic location. In the screenhouse, the AA diploid Calcutta 4 had the lowest apterous aphid density per plant (9.7 ± 4.6) compared with AAB triploid Waema, which had the highest aphid densities (395.6 ± 20.8). In the field, the highest apterous aphid density per plant (29.2 ± 6.7) occurred on the AAB triploid Batard and the lowest (0.4 ± 0.2) on the AA diploid Pisang Tongat. The AA diploid Tapo was highly susceptible to BBTD (100% infection) compared with the genotypes Balonkawe (ABB), PITA 21 (AAB), Calcutta 4 (AA), and Balbisiana Los Banos (BB), which remained uninfected. The *Musa* genotypes with apparent resistance to BBTD and least susceptibility to aphid population growth provide options for considering aphid and BBTD resistance in banana and plantain breeding programs.

## 1. Introduction

Banana (and plantain, *Musa* spp. L., Zingiberales: Musaceae) is among the top 20 food crops worldwide [1]. The cooking banana types are among the most important crops cultivated for household food security and income generation by millions of smallholder farmers under subsistence-farming conditions in sub-Saharan Africa (SSA), where they are largely grown in mixed-crop fields and backyards of household compounds. Banana productivity in SSA remains low at 7.3 million metric tonnes/ha [1], mainly because of the widespread negative impact of several endemic and exotic pests and diseases, including banana bunchy top disease (BBTD). BBTD is caused by the banana bunchy top virus (BBTV, genus Babuvirus) and is the most destructive virus disease of banana worldwide [2,3]. BBTD was first reported from Fiji in 1989 and is presently known to occur in 37 countries. In SSA, BBTD was first reported from the Democratic Republic of Congo. The virus is presently known to occur in 17 African countries (Available online: https://www.bbtvalliance.org/index.php/bbtv (accessed on 2 March 2022)) where BBTV has since emerged as a serious threat to banana production [3]. BBTV infection of banana plants results in a range of symptoms that generally culminate in a bunchy appearance at the top of severely stunted pseudostems [4]. The virus-infected plants do not produce fruit when the infection starts before flowering, while late infections result in deformed and inedible fruits [5]. Regardless of the time of infection, BBTV infection leads to 100% banana fruit yield loss from the infected plants [6,7].

BBTV is transmitted through vegetative propagation of infected banana propagules and by the banana aphid, *Pentalonia nigronervosa* Coquerel (Hemiptera: Aphididae), which occurs on plants in the Musaceae [8]. A closely related species, *Pentalonia caladii* van der Goot, frequently found on plants in the Araceae and Zingiberaceae [8,9], has been shown to transmit BBTV under experimental conditions, albeit much less efficiently than *P. nigronervosa* [10]. Considering its poor BBTV-transmission efficiency and its generally restricted distribution to non-*Musa* spp., *P. caladii* is unlikely to play a significant role in the natural transmission of BBTV in banana plantations. However, *P. caladii* could be a significant vector in BBTV transmission to other hosts in which BBTV was recently detected, including *Heliconia* sp. in Hawaii (USA) [11] and *Alpinia galanga* (L.) Willdenow and *Curcuma longa* L. in Indonesia [12].

The banana aphid transmits BBTV in a persistent, circulative, and non propagative manner [13,14], while there is no evidence for mechanical viral transmission [13,15]. The banana aphid can acquire the virus after at least 4 h of the acquisition access period (AAP) on infected tissue and requires a minimum inoculation access period (IAP) of 15 min to transmit the virus [13]. The aphid retains the virus throughout its life. BBTV-transmission efficiency by *P. nigronervosa* increases with increased AAP, IAP, virus titer in the source plant, and aphid abundance [13].

Several cultural and chemical approaches were developed for BBTD management, including the use of virus-free planting material, quarantine measures, roguing of diseased plants, and use of pesticides to control the aphid vector [16,17,18]. While these approaches were effective in large-scale monoculture banana plantations, they have not been widely adopted by smallholder farmers in SSA due to the low availability of virus-free planting materials, high costs of pesticide use for aphid control, and high labor requirement for rouging-based methods [19,20]. Host-plant resistance to the virus and/or the aphid vector, particularly in smallholder farming environments of SSA, offers the most economical and environmentally sound means for controlling virus diseases [21,22,23,24,25,26]. Previous studies focused on assessing *Musa* genotypes’ resistance against BBTD [5,27,28,29], while resistance to the banana aphid has rarely been evaluated.

This study covers the evaluation of a set of *Musa* genotypes representing all known *Musa* ploidy levels and genomic groups (Table 1) to identify resistance to BBTV and its banana aphid vector. Resistance to the banana aphid was evaluated in the absence of BBTV under a semi-controlled environment, while resistance to the aphid and BBTV was assessed over 36 months in the field under natural aphid colonization and BBTV infections. The results demonstrated the differential response of genotypes to both aphid performance and BBTD, and identified promising genotypes with high levels of tolerance to the virus vector and to the disease.

## 2. Materials and Methods

### 2.1. Planting Material

*Musa* genotypes used in this study were sourced from the international *Musa* collection held in genebanks of the International Institute of Tropical Agriculture (IITA) in Ibadan, Nigeria, the International Transit Center of Bioversity International in Leuven, Belgium, and the IITA station in Yaoundé, Cameroon (Table 1). Virus-free stocks of all sourced genotypes were propagated in vitro at the IITA tissue-culture laboratory in Yaoundé before their use in the screenhouse and field trials. Acclimatized plants were grown in 15 cm diameter pots containing a mixture of pasteurized forest topsoil, sand, and poultry manure at a ratio of 3:1:1 and maintained for 5–6 months in an insect-proof screenhouse at the IITA station in Yaoundé, Cameroon, a BBTV-free area of Cameroon.

### 2.2. Screening of Musa Genotypes for Banana Aphid Population Growth in the Screenhouse

Virus-free plants of 38 genotypes were evaluated for banana aphid growth potential under screenhouse conditions at the IITA-Cameroon campus in Yaoundé (03°51.839′ N/011°7.748′ E, ~770 m.a.s.l.) (Table 1). Banana aphids of mixed stages (nymphs and adults) were collected from banana plants at the IITA-Cameroon experimental farm to establish an aphid colony on virus-free potted plants of the cultivar ‘Williams’ (AAA). The plants were raised for about 55 days (approximately three generations) in an insect-proof screenhouse before their use. *Pentalonia nigronervosa* identity was established by examining morphological features of at least 20 aphids under a phase-contrast microscope [30]. Twelve plants per genotype arranged in three replicates of four plants each were evaluated. Using a fine camel-hair brush, five 4th instar aphids from stock cultures were gently teased-off the plant and transferred onto an unfurled top leaf of each test plant. All plants of the 38 genotypes were infested with aphids collected from the stock colony on the same day. The infested plants were maintained in an insect-proof cage (70 × 50 × 43 cm). The experiment was conducted from July to September 2013. Aphid census on the whole plant started one week after the transfer of aphids and continued weekly for nine weeks. Plants were taken out of the cage for a brief period for a census of aphids. Apterous and alate aphids were tallied separately. Test plants were watered as needed. Temperature and relative humidity within the screenhouse were monitored with a Hobo Pro v2 logger (Onset Computer Corporation, Bourne, MA, USA) suspended just above the cages in the center of the screenhouse. Temperatures during the experiment fluctuated between 18.4 and 36.2 °C with an average of 23.0 ± 0.04 °C. Relative humidity ranged from 37.7 to 99.4%, with an average of 85.8 ± 0.2%. The photoperiod was 12:12 L:D (±30 min).

### 2.3. Evaluation of Musa Genotypes against Banana Aphid and BBTD under Field Conditions

The field trial was established in Abang Minko’o (02°19.513′ N/011°26.362′ E, 563 m.a.s.l.), an area in the South Region of Cameroon where BBTD is endemic [6]. BBTD is widely prevalent in the area, with 94.3% of fields with one or more BBTD-infected plants and a within-field disease incidence ranging between 0.17% and 36% (Ngatat et al., unpublished data). The nearest banana farms were at 30, 15, 24, and 1000 m from the field borders in the north, east, south, and west, respectively. This area of the field experiment is located in a humid forest zone with bimodal rainfall. Temperature and relative humidity were monitored with Hobo^®^ Pro v2 logger (Onset Comp., Bourne, MA, USA) installed under a waterproof shelter, while rainfall data was collected with a Tru-Chek™ rain gauge (Edwards Man. Co., Albert Lea, MN, USA) placed in an open area. Temperatures in the experimental field ranged from 14 to 37 °C with an average of 24.1 ± 0.02 °C, while relative humidity ranged from 35 to 100%, with an average of 89.1 ± 0.07%. Average yearly rainfall for the 3 years was 1806 ± 122 mm.

Virus-free, hardened tissue-culture plants of 44 genotypes, including 34 genotypes evaluated for aphid population growth under the screenhouse experiment, were planted at 2 × 2 m row spacing in holes of 30 × 30 × 30 cm. One kg of poultry manure (2.1, 1.3, and 0.8% N, P, K, respectively) was mixed with soil at planting time in September 2013. The experiment was terminated 36 months after its initiation. The experimental layout followed a complete randomized block design with three blocks (=replicates), with an area of 704 m^2^ per block and a 4 m distance between blocks. Within each block, all genotypes were planted in plots of three rows and five plants per row for a total of fifteen plants per plot. All plants were free of banana aphids and BBTD at planting. To facilitate and augment the natural spread of the virus, 176 BBTD-symptomatic suckers, obtained from the virus-affected banana fields in the vicinity of the experimental field plot, were planted at the edges of rows between blocks [29]. Aphid infestation and BBTV infection of experimental plants occurred naturally. The field was weeded manually. Dead leaves were pruned as needed. The field was managed under rainfed conditions with no additional inputs during the 36 months of the experiment.

**Figure 1:**
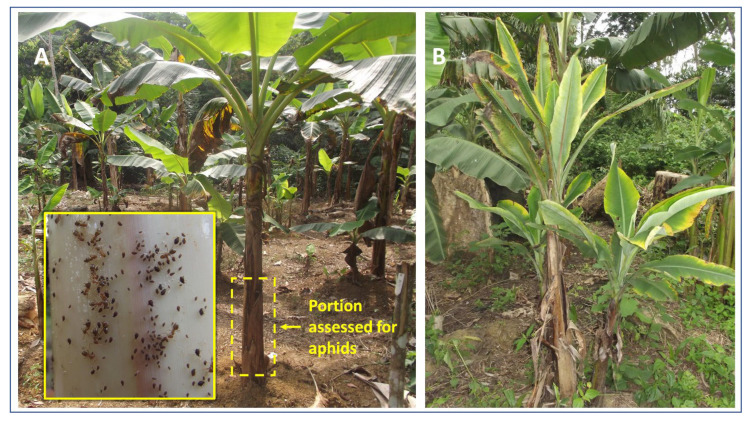
(**A**) The lower part of the pseudostem of a banana shoot used for aphid counting in the field trial; inset: banana aphids on pseudostem. (**B**) *Musa* plant with typical bunchy top disease symptoms in the field.

Census for banana aphid and BBTD-affected plants were initiated 2 months after planting (MAP) and continued monthly for 36 months. The number of banana aphids (all life stages) was counted on the bottom third of the oldest pseudostem on three plants per replicate (Figure 1) [31,32]. After flowering and bunch production of the oldest pseudostem, aphid census was shifted to the next oldest sucker of the same mat [29]. Occasionally, aphid occurrence was observed on the throat and leaves of the census plants, but they were not counted to maintain the standard assessment to only the lower portion of the pseudostem. BBTD incidence was recorded at the same time of aphid census. All the shoots (pseudostems) of a mat (=plant) in a replicate were carefully checked for the presence of BBTD symptoms. A mat was counted as symptomatic based on typical BBTV symptoms on at least one pseudostem. The time between planting and the first appearance of BBTD symptoms on a plant was evaluated for each genotype. At the end of the experiment (i.e., 36 MAP), leaf samples of all asymptomatic plants were collected for BBTV testing by polymerase chain reaction (PCR) as described previously [6]. Estimation of the number and weight of bunches at harvest time was not possible due to thefts.

### 2.4. Data Analysis

Aphid abundance, including apterous and alate forms, was estimated for each genotype present in screenhouse and field trials. The area under the infestation pressure curve (AUIPC), which represents the increase in aphid population over time, was computed for each genotype using the modified formula from Shaner and Finney [33]. $\mathrm{AUIPC}=\overset{n-1}{\underset{i=1}{\sum }}(\frac{y_{i}+y_{i+1}}{2})\times (t_{i+1}-t_{i})$ where *i* = the ranking of the assessment, *n* = number of days between *i* and *i* + 1 assessment, and *y* = number of insects at time *t*. BBTD incidence- based on symptom observation in the field of each genotype was calculated as $\mathrm{BBTD}\;incidence=\frac{Total\;mats\;infected}{Total\;mats\;planted}\times 100.\;$A quantitative summary of disease over time represented by the area under the disease progress curve (AUDPC) was computed for each genotype using the formula from Shaner and Finney [33]. $\mathrm{AUDPC}=\overset{n-1}{\underset{i=1}{\sum }}(\frac{y_{i}+y_{i+1}}{2})\times (t_{i+1}-t_{i})$, where *y_i_* is the proportion of infected plants at the *i*th observation, *t_i_* is time in days at the *i*th observation, and *n* is the total number of observations. Virus incidence, based on virus-positive leaves by PCR analysis, was calculated as the percentage of infected leaf samples for each genotype. All response variables, i.e., apterous aphids, alate aphids, BBTD incidence, and AUDPC, were tested for normal distribution using the Shapiro–Wilk test at *p* > 0.05 [34]. Response variables that were not normally distributed were analyzed with the Kruskall–Wallis nonparametric test. Bonferonni pairwise comparison of genotypes was used because of the large number of pairwise comparisons.

To select the genotypes that were most or least susceptible to banana aphid and/or to BBTD, in each genomic group for both screenhouse and field trials, four genotypes with an extreme mean (two highest and two lowest) aphid abundance or BBTD incidence were selected and compared. Correlation analysis was used to relate aphid densities in the screenhouse to those in the field on each of the 34 genotypes present both in the screenhouse and in the field. To rank the genotypes, while simultaneously considering their reaction under natural conditions to both banana aphid and BBTD, a heatmap with hierarchical clustering was used for classification based on both AUDPC and AUIPC values for each genotype obtained from the field trial. All statistical analyses were performed with R 3.6.2 package (R Development Core Team), while the heatmap was generated with JMP 8.2 (SAS Institute).

## 3. Results

### 3.1. Banana Aphid Abundance on Musa Genotypes in the Screenhouse

Apterous aphid densities were higher on triploid (155.3 ± 21.4) and tetraploid (139.7 ± 13.7) than on diploid (74. 8 ± 10.5) genotypes (χ^2^ = 14.7, df = 2, *p* < 0.001). Alate aphid densities were also greater on triploid (7.4 ± 1.4) and tetraploid (5.3 ± 1.4) than on diploid (1.4 ± 0.4) genotypes (χ^2^ = 13.6, df = 2, *p* = 0.001). A difference was observed among diploid genotypes for apterous aphid abundance (χ^2^ =17.7, df = 10, *p* = 0.006) but not for alate aphids (χ^2^ = 13.98, df = 10, *p* = 0.17) (Table 2). However, both apterous and alate aphid abundance differed among triploid genotypes (χ^2^ = 23.4, df = 14, *p* = 0.04 and χ^2^ = 32.2, df =14, *p* = 0.004, respectively). The highest aphid density was observed on the Waema (AAB), while the lowest density was observed on Ice cream (ABB) (Table 3). The highest alate density was observed on Ebang (AAB), while the lowest occurred on Yangambi Km5 (AAA). Similarly, both apterous and alate aphid abundance differed among genotypes in the tetraploids (χ^2^ = 21.5, df = 1, *p* = 0.03 and χ^2^ = 25.2, df = 11, *p* = 0.009, respectively) (Table 4). The highest apterous and alate aphid densities were observed on FHIA 03 (AABB), while the lowest occurred on T6 (AAAA). Comparison of two genotypes with highest and lowest aphid densities from each ploidy level showed significant differences among the sorted genotypes for apterous aphid (χ^2^ = 30.0, df = 11, *p* = 0.002) and alate aphid (χ^2^ = 30.1, df = 11, *p* = 0.002) densities. Apterous aphid density was highest on Waema (AAB) and lowest on Calcutta 4 (AA), while alate aphid density was highest on Ebang (AAB) and lowest on Calcutta 4 (AA) (Table 5).

### 3.2. Banana Aphid and BBTD Occurrence on Musa Genotypes under Field Conditions

#### Abundance of Banana Aphid in the Field

In the field trial, there were also large differences among ploidy levels in apterous aphid densities, but, not for alate aphid densities (χ^2^ = 22.9, df = 2, *p* < 0.001 and χ^2^ = 5.6, df = 2, *p* = 0.06, respectively). After 36-months, apterous aphid densities were similar on triploids (10.7 ± 1.4) and tetraploids (10.7 ± 1.3). In case of the diploid genotypes, differences were observed among genotypes for apterous aphid densities (χ^2^ = 15.8, df = 8, *p* = 0.04) but not for alate aphids (χ^2^ = 12.1, df = 8, *p* = 0.15). The highest apterous aphid density was on Chuoi Man (AB), while the lowest was on Pisang Tongat (AA) (Table 6). In the triploid group, both apterous and alate aphid abundance differed among genotypes (χ^2^ = 40.5, df = 18, *p* = 0.002, χ^2^ = 39.9, df = 18, *p* = 0.002, respectively). The highest apterous aphid density was recorded on Batard (AAB), while the lowest was recorded on PITA 24 (AAB). On the other hand, alate aphid densities were considerably lower than apterous aphids on all the genotypes, and the highest density of alates was recorded on Essong (AAB), while the lowest was on Lep Chang Kut (BBB) (Table 7). In the tetraploids, significant differences were observed between genotypes for apterous aphid densities (χ^2^ = 34.8, df = 15, *p* = 0.003) but not for alate aphid densities (χ^2^ = 20.6, df = 15, *p* = 0.15); the highest apterous aphid density was on CRBP 39 (AAAB), while the lowest density was on CRBP 37 (AAAB) (Table 8). Analysis performed on 12 genotypes to sort out the best and the worst genotypes for aphid performance under field conditions, with two from each ploidy group with highest aphid densities and two with lowest aphid densities, showed large differences among the sorted genotypes for apterous aphid densities (χ^2^ = 27.7, df = 11, *p* = 0.004) and alate aphid densities (χ^2^ = 20.5, df = 11, *p* = 0.02). The highest apterous aphid densities were observed on Batard (AAB) and the lowest on Pisang Tongat (AA). The highest alate aphid densities were recorded on Essong (AAB) and the lowest were on Yagambi km5 (AAA) (Table 9).

### 3.3. Time to First BBTD Symptoms

On diploids, the genotype Chuoi Man (AB) presented the first BBTD symptoms at 2 MAP while it took 36 months to observe the first symptoms on Ney Poovan (AB) (Table 6). On triploids, the first BBTD symptoms were observed at 2 MAP on Waema (AAB), 3 MAP on Williams (AAA), and PITA 23 (AAB), while symptoms were observed only at 36 MAP on Fougamou (ABB) (Table 6). On tetraploids, BBTD symptoms were first observed at 3 MAP the AAAA genotypes Buccaneer, SH 3436-9, T6 (AAAA) and on FHIA 03 (AABB), and the latest at 26 MAP on CRBP 37 (AAAB) and 28 MAP on CRBP 535 (AAAB) (Table 7). Four genotypes—Calcutta 4 (AA), Balbissiana Los Banos (BB), PITA 21 (AAB), and Balonkawe (ABB)—did not show any BBTD symptoms at any time during the 36 months of the experiment.

Overall, latent BBTV infection was detected by PCR assays in asymptomatic plants of 13 genotypes, all ploidy considered at 36 MAP at the end of the experiment. Of the dipoids, BBTV was detected in 15.6% of the 45 leaf samples from Kunnan (AB), while the virus was not detected in any of the asymptomatic plants of other diploid genotypes (Table 6). On the triploid genotypes, the highest virus incidence (16.7% for total 6 plants tested) was detected in Williams (AAA); BBTV was not detected in the asymptomatic plants of Yagambi Km5 (AAA), the AAB genotypes (Batard, Ebang, Elat, Essong, Waema), Fougamou (ABB), Balonkawe (ABB), and Lep Chang Kut (BBB) (Table 7). On the tetraploids, 17.8% virus incidence (15 plants tested) was observed on SH 3436-6 (AAAA), while no virus was detected on the asymptomatic leaves of 12 genotypes, including the AAAA genotypes (BITA 2, Buccaneer, FHIA 23, T6), the AAAB (BITA 8, CRBP 37, CRBP 535, CRBP 568, FHIA 21, IRFA 908), FHIA 03 (AABB) (Table 8).

### 3.4. BBTD Incidence and Area under Disease Progress Curve (AUDPC)

No significant difference was observed among the ploidy levels for the disease incidence (df = 2, χ^2^ = 0.7, *p* = 0.7) and AUDPC (df = 2, χ^2^ = 0.9, *p* = 0.7). In the diploid group, significant differences were observed among genotypes for both BBTD incidence (χ^2^ = 19.6, df = 8, *p* = 0.01) and AUDPC (χ^2^ = 20.2, df = 8, *p* = 0.009). At 36 MAP, Tapo (AA) was the genotype with highest disease incidence—100% of the plants expressed BBTD symptoms. Tapo also had the highest AUDPC, which together with its disease incidence indicate that Tapo was the most susceptible genotype to BBTD. On the contrary, none of plants of the wild genotypes Calcutta 4 (AA) and Balbisiana Los Banos (BB) showed any BBTD symptoms at any time during the experiment (Table 6), which along with their virus-free status at 36 MAP indicate that these two genotypes are resistant to BBTD—at least for the duration of our experiment. In the triploids, significant differences were similarly observed among genotypes for BBTD incidence (χ^2^ = 32.2, df = 18, *p* = 0.02) and AUDPC (χ^2^ = 34.9, df = 18, *p* = 0.01); 66.7 % of plants of FHIA 25 (AAB) expressed BBTD symptoms, while no disease was observed on Balonkawe (ABB) and PITA 21 (AAB). Moreover, the highest AUDPC was observed on Yangambi Km5 (AAA) and FHIA 25, while the lowest was recorded on both Balonkawe (ABB) and PITA 21 (AAB) (Table 7). In the tetraploid group, there was no difference among genotypes for both disease incidence (χ^2^ = 19.5, df = 15, *p* = 0.2) and AUDPC (χ^2^ = 18.7, df = 15, *p* = 0.2) (Table 8).

Analysis performed on 12 genotypes sorted from the four divergent genotypes of each ploidy group (four from each ploidy group, two with highest AUDPC, and two with lowest AUDPC) showed significant differences among the sorted genotypes for disease incidence (χ^2^ = 26.9, df = 11, *p* = 0.005) and AUDPC (χ^2^ = 28.5, df = 11, *p* = 0.003), the highest disease incidence and AUDPC was recorded on the AA diploid Tapo, while the lowest incidence and AUDPC were recorded on Calcutta 4 (AA), Balbisiana Los Banos (BB), Balonkawe (ABB) and PITA 21 (AAB) (Table 10). Generally, at the field level, average BBTD incidence was 15.3, 26.1, and 34.1%, respectively, at 12, 24, and 36 MAP.

### 3.5. Classification of Musa Genotypes for the Response to Banana Aphid and BBTD in Field 

**Figure 2:**
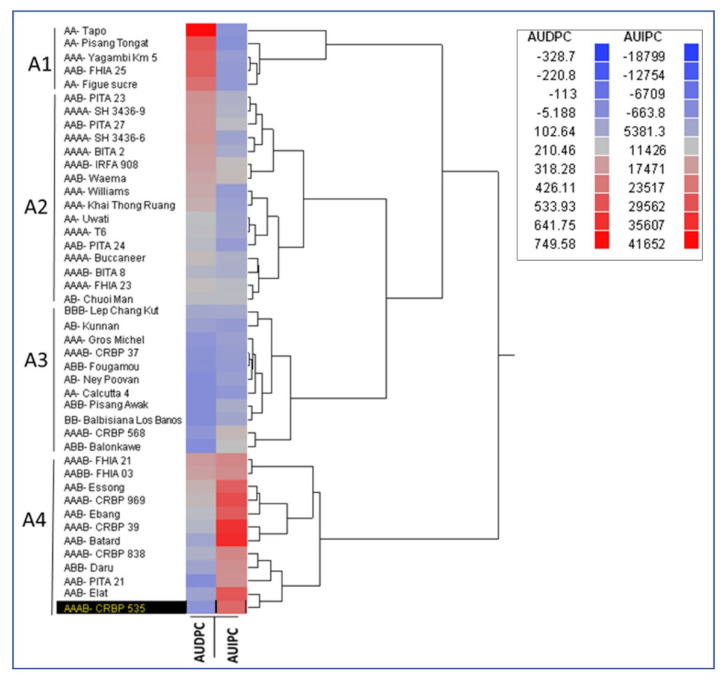
Heatmap and hierarchical cluster analysis dendrogram of the 44 *Musa* genotypes evaluated based on area under disease pressure-curve (AUDPC) values of banana bunchy top disease (BBTD) and area under infestation progress-curve (AUIPC) values of banana aphids (*Pentalonia nigronervosa*) obtained after 36 months of evaluation in a field trial. The range of AUDPC and AUIPC values is given in the inset. Accessions in the A1 cluster are highly susceptible to BBTD and least preferred by banana aphids, accessions in the A4 cluster are highly susceptible to banana aphids, and accessions in the A3 cluster are resilient to BBTD and banana aphids.

The heatmap with hierarchical cluster showed that genotypes were clustered into four groups: A1, A2, A3, and A4 (Figure 2). All the five genotypes in group A1 were highly susceptible to BBTD and least susceptible to the banana aphid, including Figue Sucree (AA), Pisang Tongat (AA), Yangambi Km5 (AAA), and FHIA 25 (AAB). Group A2 included 18 genotypes of different genomic compositions, from moderate susceptibility to both BBTD and the banana aphid. Group A3 included 11 genotypes of different genomic compositions with least or slight susceptibility to both BBTD and banana aphid. Group A4 included 10 genotypes slightly susceptible to BBTD and highly susceptible to the banana aphid (Figure 2).

### 3.6. Correlation for Aphid Performance in Screenhouse and Field

There was a positive and moderately high correlation between screenhouse and field aphid densities on each of the 34 genotypes present both in the screenhouse and the field (r = 0.529, *p* = 0.001). The genotypes with the highest aphid densities, in both screenhouse and field trials, were from the AAB genomic group followed by the AAAB group, while the lowest aphid densities occurred on the AA genomic group in both the screenhouse and the field (Table S1).

## 4. Discussion

Most banana cultivars originated from intraspecific or interspecific hybridization between wild diploid *M. acuminata* (A-genome, 2*n* = 22) and *M. balbisiana* (B-genome, 2*n* = 22) species of section *Eumusa*, including diploid (AA, BB and AB), triploid (AAA, AAB and ABB), and tetraploid (AAAB, AABB, ABBB) variants [35]. This study showed that in the screenhouse and the field trials, there was a wide variation in the performance of banana aphids on the *Musa* genotypes with different A and B genome composition and ploidy levels. In the screenhouse, aphid densities on triploid and tetraploid genotypes were higher than on diploid genotypes. The rates of aphid population growth were faster and reached higher densities on two AAB triploids, Waema and Ebang, than any other genotype. Aphid densities under field conditions were relatively lower than in the screenhouse, but the trend of banana aphid performance on *Musa* genotypes was similar under both screenhouse and field conditions. For instance, the AAB triploids Batard, Ebang, Essong, and Elat and the AAAB tetraploid hybrids CRBP 39, CRBP 969, and CRBP 535 were highly suitable to banana aphid population growth, resulting in the highest aphid densities both under field and screenhouse conditions. Aphid densities were lowest (about 10-fold lower population) on AA diploid genotypes Pisang Tongat, Figue Sucree, and Calcutta 4 compared with AAB and AAAB genotypes under field conditions. In general, aphids were more abundant on triploid and tetraploid genotypes combining both A and B genomes (AAB, AAAB) than on those combining only A (AA or AAA) or only B (BB and BBB) genomes. Only one genotype, Yawa 2, evaluated in this study corresponds to the ABBT group, which is a natural cross of section *Musa* (*Eumusa*) (AA and BB) × section *Australimusa* (TT). This genotype under screenhouse evaluation supported high densities of banana aphids, but the genotype was not assessed under field conditions due to insufficient planting material.

Further studies are necessary to understand the underlying factors contributing to the differential aphid establishment rates in relation to host genomic composition. One aspect of investigation should focus on the thickness of epicuticular wax on leaf- petiole, and pseudostem. For instance, a thick epicuticular wax was reported to increase resistance to black Sigatoka of banana [36]. Several studies have shown that successful aphid colonization and performance are affected by multiple factors, including (i) chemical content of the sap (e.g., nitrogen and carbon levels, and free-amino-acid composition in sap) [37,38]; (ii) defensive compounds that reduce aphid feeding and multiplication rate [39]; (iii) plant physical properties, which serve as barriers to feeding and growth (e.g., leaf pubescence, smoothness or roughness of leaves, the presence of trichomes or the shape and color of the leaves) [40]; (iv) leaf and plant color, which affect attractiveness and landing behavior [41]; and (v) chemical cues affecting landing decision [42,43]. Further studies should consider comparisons of the physical and chemical properties of *Musa* genotypes supporting low and high aphid population densities to understand the mechanisms contributing differential rate of establishment and population growth on different *Musa* genotypes evaluated in this study.

As observed for most aphid species, apterous banana aphids were more abundant than alates in both the screenhouse and the field. Owing to their higher mobility, alates play a major role in the horizontal transmission of BBTV within and between the fields. Alate abundance is generally linked to increasing density of apterous forms. Consequently, a *Musa* genotype that supports the establishment of high densities of banana aphids poses an increased risk for the horizontal spread of BBTV and heightens virus spread in the field. In the field trial of this study, *Musa* genotypes with high BBTD incidence did not support relatively high numbers of alate aphids, and the genotypes with high alate populations were moderately affected by BBTV. Plant viruses are known to induce specific changes in the host plant, modifying the behavior of its vector, which may favor or impair virus transmission. A similar observation was made in a recent study that demonstrated differential emissions of volatile organic compounds (VOCs) by healthy and BBTV-infected banana plants of Williams (AAA) and a Pacific triploid (AAB) plantain [42]. Relatively higher VOCs detected in the BBTV-infected plants were attributed to a stronger attraction to alate and apterous banana aphids than in uninfected plants [42]. The diversity and concentration of VOCs were greater in the AAB plantain than in AAA Williams, which implies differential production of VOCs depending on the genomic composition. 

The *Musa* genotypes evaluated in the field showed large variations in BBTD incidence ranging from 0 to 100%. BBTD expression varied significantly with *Musa* genotypes, with the highest AUDPC on the AA diploid Tapo, which conversely was among the genotypes with low aphid densities. This was followed by two AA diploids, Pisang Tongat and Figue Sucree; and two triploids, FHIA 25 (AAB) and Yangambi Km 5 (AAA). Generally, genotypes with only A genome (AA and AAA genomic groups) were more susceptible to BBTV infection, except for the wild diploid Calcutta 4 (AA), which was not infected after 36 months of exposure in the field, compared with genotypes with B genome. In regard to the genotypes with both A and B genomes, triploids can be found throughout the BBTV susceptibility spectrum; for example, the triploid FHIA 25 (AAB) was highly susceptible while PITA 21 (AAB) and Balonkawe (ABB) remained uninfected after 36 MAP in the field. In general, genotypes with more than one copy of the B genome (BB, BBB, ABB, and AABB) showed less susceptibility (0 to <20% incidence) to BBTV infection. For instance, low infection was recorded on the ABB triploids Daru, Pisang Awak, Fougamou, and BBB triploid, Lep Chang Kut. No infection was recorded on the wild diploid Balbisiana Los Banos (BB) and the triploid Balonkawe (ABB). This leads to the hypothesis that *Musa* genotypes with two or more copies of the B genome possess better tolerance to BBTV infection. However, the exception was the synthetic hybrid FHIA 03 (AABB), which despite having two sets of BB chromosomes showed a relatively high BBTD incidence (62.5%). Another genotype, the synthetic hybrid PITA 21 (AAB), despite having only one copy of the B chromosome, showed a very high tolerance to BBTV infection. *Musa* genomic studies can shed light on the potential role of the B genome in banana resistance to BBTD. The observations on BBTD occurrence on some of the genotypes in this study corroborate with previous studies [28,29].

Wild genotypes often harbour some traits linked to resistance to disease and/or pests, as observed in the present studies with Balbisiana Los Banos (BB) and Calcutta 4 (AA). The diploid Calcutta 4 is known to be resistant to black leaf streak disease (BLSD) caused by *Mycosphaerella fijiensis* [44]. Calcutta 4 (AA) has been used extensively in *Musa* breeding as a source of black Sigatoka and BLSD resistance [44] and for its partial resistance to banana weevil [45]. Calcutta 4 has also been reported as resistant to some races of *Fusarium oxysporum* f. sp. *cubense* in subtropical Australia [46], and *M. balbisiana* accessions have shown resistance to Xanthomonas wilt in a greenhouse trial [47]. The B genome is also known to confer some drought resistance in *Musa* genotypes [48]. The four genotypes—Calcutta 4 (AA), Balbisiana Los Banos (BB), PITA 21 (AAB), and Balonkawe (ABB)—that showed high tolerance to BBTV infection are of interest for breeding programs. PITA 21, a plantain hybrid developed by IITA and resistant to BLSD, is among hybrids grown by farmers in at least four countries in Africa, including Cameroon and Nigeria [49], where BBTD is present. Balonkawe is a traditional landrace widely used in the Philippines. Both PITA 21 and Balonkawe can be used to broaden sources of resistance to BBTV. However, further research is necessary to assess the robustness of resistance by experimental inoculation of these plants with viruliferous aphids under controlled conditions.

The grouping based on reaction to banana aphid and BBTD identified a group of five genotypes that are highly susceptible to BBTD and less susceptible to the banana aphid, including Tapo, Pisang Tongat, Figure sucre, Yagambi Km5, and FHIA 25. The second group of 10 genotypes, Batard, Essong, Ebang, Elat, PITA 21, CRBP 39, CRBP 535, CRBP 838, CRBP 969, and Daru, were less susceptible to BBTD and highly susceptible to the banana aphid. These groupings indicate that BBTD incidence is a genotype trait and is not positively related to aphid abundance on a genotype [29]. Although aphids were found on PITA 21 (AAB), Balonkawe (ABB), Calcutta 4 (AA), and Balbisiana Los Banos (BB), these genotypes were free of BBTV at 36 MAP in a BBTV endemic area. The lack of BBTV infection on these four genotypes could result from cases where aphids fed on them may not have been viruliferous, or the plants were difficult to infect by aphid inoculation. However, considering the establishment of the trial in a BBTV hotspot, with high levels of inoculum in the vicinity and the presence of spreader plants (BBTV symptomatic plants), and the same banana aphid population moving randomly in the field, there is a high probability that viruliferous aphids would have spread onto the plants of these four genotypes. However, due to evidently high tolerance, the plants remained uninfected. These genotypes may be resistant to virus inoculation. Hooks et al. [5] stated that despite susceptibility to BBTV, some banana cultivars have some resistance to virus inoculation by the banana aphid. Further experimental inoculation with viruliferous aphids is important to understand the reason for the uninfected status of these genotypes, even after prolonged exposure to BBTV in an endemic location.

## 5. Conclusions

*Musa* genotypes evaluated in this study exhibited differential reactions to the banana aphid under screenhouse and field conditions. Aphid densities were higher on triploid and tetraploid genotypes containing both A and B genomes than those combining only A (AA or AAA) or B (BB and BBB) genomes. Similarly, genotypes responded differently to BBTD. In general, *Musa* genotypes with two copies of the B genome showed high tolerance to BBTV, and none of the plants of four genotypes [PITA 21 (AAB), Balonkawe (ABB), Calcutta 4 (AA), and Balbisiana Los Banos (BB)] were infected by BBTV after 36 months of exposure to BBTV inoculum in the field. These genotypes could be used as a source of resistance for breeding BBTV-resistant hybrids. Further studies are necessary to elucidate the underlying mechanisms contributing to the reduced susceptibility to banana aphid and BBTV.

## Figures and Tables

**Table 1:** The list of *Musa* genotypes evaluated against BBTV and banana aphid, *Pentalonia nigronervosa*.

Ploidy Level	Genomic Group	Genotype	Source
Diploid	AA	Calcutta 4	ITC
		Figue Sucrée	ITC
		Pisang Tongat	ITC
		Uwati	ITC
		Tapo	ITC
	AB	Ney Poovan	ITC
		Auko *	ITC
		Vunapope *	ITC
		Kunnan	ITC
		Chuoi Man	ITC
	BB	Balbisiana Los Banos	ITC
Triploid	AAA	Gros Michel	ITC
		Khai Thong Ruang	ITC
		Yangambi Km5	ITC
		Williams	IITA
	AAB	Batard ^#^	IITA
		Ebang	IITA
		Elat ^#^	IITA
		Essong	IITA
		FHIA 25	IITA
		PITA 21	IITA
		PITA 23	IITA
		PITA 24 ^#^	IITA
		PITA 27 ^#^	IITA
		Waema	ITC
	ABB	Ice cream *	ITC
		Pisang Awak	ITC
		Balonkawe	ITC
		Daru	ITC
		Fougamou ^#^	IITA
	BBB	Lep Chang Kut	ITC
Tetraploid	AAAA	T6	ITC
		Buccaneer	ITC
		SH3436-9	ITC
		SH3436-6	ITC
		FHIA 23	IITA
		BITA-2	ITC
	AAAB	BITA 8 ^#^	IITA
		IRFA 908	ITC
		FHIA-21	ITC
		CRBP 37	ITC
		CRBP 39	ITC
		CRBP 568 ^#^	CARBAP
		CRBP 535 ^#^	CARBAP
		CRBP 838 ^#^	CARBAP
		CRBP 969 ^#^	CARBAP
	AABB	FHIA-03	ITC
	ABBT	Yawa 2 *	ITC

* Genotypes evaluated only in the screenhouse experiment. ^#^ Genotypes evaluated only in the field experiment. IITA = International Institute of Tropical Agriculture; ICT = International Transit Center; CARBAP = African Center for Banana and Plantain Research.

**Table 2:** Apterous and alate banana aphid abundance (mean ± SE) on diploid genotypes for nine weeks after experimental infestation under screenhouse conditions.

Genomic Group	Genotype	Apterous Aphid Abundance	Alate Aphid Abundance
AA	Calcutta 4	9.7 ± 4.6 ^a^	0 ^a^
AA	Figue sucrée	120.9 ± 35.5 ^a^	1.3 ± 0.2 ^a^
AA	Pisang Tongat	73.2 ± 16.6 ^a^	2.1 ± 1.0 ^a^
AA	Tapo	58.4 ± 20.6 ^a^	4.1 ± 3.3 ^a^
AA	Uwati	82.8 ± 28.9 ^a^	1.0 ± 0.6 ^a^
AB	Auko	118.3 ± 48.4 ^a^	0 ^a^
AB	Chuoi Man	40.3 ± 19.9 ^a^	0.2 ± 0.2 ^a^
AB	Kunnan	136.7 ± 66.6 ^a^	2.9 ± 1.4 ^a^
AB	Ney Poovan	17.9 ± 7.5 ^a^	0.1 ± 0.0 ^a^
AB	Vunapope	67.4 ± 23.8 ^a^	2.8 ± 2.1 ^a^
BB	Balbisiana	111.4 ± 18.4 ^a^	0.2 ± 0.2 ^a^
Chisq		17.66	13.9
df		10	10
*p*		0.06	0.17

Means with the same letter in a column are not significantly different (Bonferroni test, *p* = 0.05).

**Table 3:** Apterous and alate banana aphid abundance (mean ± SE) on triploid genotypes for nine weeks after experimental infestation under screenhouse conditions.

Genomic Group	Genotype	Apterous Aphid Abundance	Alate Aphid Abundance
AAA	Gros Michel	115.6 ± 55.8 ^ab^	2.9 ± 2.8 ^ab^
AAA	Khai Tang Wang	86.7 ± 34.8 ^ab^	1.0 ± 0.8 ^ab^
AAA	William	79.5 ± 18.3 ^ab^	2.7 ± 1.4 ^ab^
AAA	Yangambi Km5	80.3 ± 9.3 ^ab^	0 ^b^
AAB	Ebang	366.1 ± 24.7 ^ab^	34.7 ±11.7 ^a^
AAB	Essong	262.3 ± 49.3 ^ab^	22.0 ± 8.4 ^a^
AAB	FHIA 25	138.9 ± 43.9 ^ab^	2.1 ± 1.2 ^ab^
AAB	PITA 23	96.8 ± 13.6 ^ab^	2.6 ± 0.6 ^ab^
AAB	Waema	521.0 ± 126.0 ^a^	31.8 ± 8.1 ^a^
ABB	Daru	114.2 ± 43.3 ^ab^	6.5 ± 5.2 ^ab^
ABB	Fougamou	124.9 ± 56.1 ^ab^	3.4 ± 2.0 ^ab^
ABB	Ice Cream	63.5 ± 5.9 ^b^	0.5 ± 0.3 ^ab^
ABB	Pisang Awak	117.9 ±25.7 ^ab^	6.8 ± 3.0 ^ab^
ABB	Balonkawe	97.7 ± 44.1 ^ab^	0.3 ± 0.3 ^b^
BBB	Lep Chang Kut	64.6 ± 22.0 ^ab^	0.1 ± 0.0 ^b^
Chisq		24.38	32.22
df		14	14
*p*		0.04	0.004

Means with the same letter in a column are not significantly different (Bonferroni test, *p* = 0.05).

**Table 4:** Apterous and alate banana aphid (mean ± SE) on tetraploid genotypes for 9 weeks after experimental infestation under screenhouse conditions.

Genomic Group	Genotype	Apterous Aphid Abundance	Alate Aphid Abundance
AAAA	BITA 2	190.4 ± 34.6 ^ab^	8.0 ± 4.4 ^ab^
AAAA	Buccaneer	153.0 ± 35.6 ^ab^	2.5 ± 0.5 ^ab^
AAAA	FHIA 23	101.6 ± 38.9 ^ab^	1.3 ± 1.0 ^b^
AAAA	SH 3436-6	117.0 ± 14.6 ^ab^	0.3 ± 0.1 ^b^
AAAA	SH 3436-9	90.0 ± 34.8 ^ab^	0.6 ± 0.4 ^b^
AAAA	T6	50.7 ± 11.0 ^b^	0.3 ± 0.2 ^b^
AAAB	CRBP 37	82.1 ± 7.8 ^b^	0.6 ± 0.4 ^b^
AAAB	CRBP 39	115.5 ± 18.2 ^ab^	3.9 ± 1.8 ^ab^
AAAB	FHIA 21	167.2 ± 55.1 ^ab^	5.6 ± 2.7 ^ab^
AAAB	IRFA 908	153.1 ± 25.2 ^ab^	7.4 ± 3.9 ^ab^
AABB	FHIA 03	327.0 ± 40.1 ^a^	27.3 ± 6.6 ^a^
ABBT	Yawa 2	128.8 ± 19.4 ^ab^	5.6 ± 2.5 ^ab^
Chisq		21.49	25.19
df		11	11
*p*		0.03	0.009

Means with the same letter in a column are not significantly different (Bonferroni test, *p* = 0.05).

**Table 5:** Apterous and alate banana aphid abundance (mean ± SE) on the 12 *Musa* genotypes sorted from ploidy groups 9 weeks after experimental infestation under screenhouse conditions.

Genomic Group	Genotypes	Apterous Aphid	Alate Aphid
Genotypes with high aphid densities			
AAB	Waema	395.6 ± 20.8 ^a^	24.9 ± 7.1 ^a^
AAB	Ebang	366.1 ± 24.7 ^ab^	34.7 ± 11.7 ^a^
AABB	FHIA 03	327.0 ± 40.1 ^ab^	27.3 ± 6.6 ^a^
AAAA	BITA 2	190.4 ± 34.6 ^abc^	8.0 ± 4.4 ^ab^
AB	Kunnan	136.7 ± 66.7 ^bcd^	2.9 ± 1.4 ^abc^
AA	Figue sucrée	120.9 ± 35.5 ^abcd^	1.3 ± 0.2 ^abc^
Genotypes with low aphid densities			
AAAB	CRBP 37	82.1 ± 7.8 ^bcde^	0.6 ± 0.4 ^bcd^
BBB	Lep Chang Kut	64.6 ± 22.0 ^cde^	0.1 ± 0.0 ^cd^
ABB	Ice Cream	63.5 ± 5.9 ^cde^	0.5 ± 0.3 ^bcd^
AAAA	T6	50.7 ± 11.0 ^cde^	0.3 ± 0.2 ^bcd^
AB	Ney Poovan	17.9 ± 7.6 ^de^	0.1 ± 0.0 ^cd^
AA	Calcutta 4	9.7 ± 4.6 ^e^	0 ^d^
Chisq		30.0	30.1
Df		11	11
*p*		0.002	0.002

Means with the same letter in a column are not significantly different (Bonferroni test, *p* = 0.05).

**Table 6:** Apterous and alate banana aphid abundance, banana bunchy top disease (BBTD) incidence, area under disease progress curve (AUDPC) and virus incidence on the diploid *Musa* genotypes after 36 months in the field.

Genomic Group	Genotype	Total Plants Planted	Month to First Symptoms	Apterous Aphid Abundance	Alate Aphid Abundance	BBTD Incidence	AUDPC	Total Tested for BBTV	BBTV Incidence (%)
AA	Calcutta 4	9	NS	1.4 ± 0.4 ^bcd^	0.2 ± 0.1 ^a^	0 ^c^	0 ^c^	7	0
AA	Figue sucree	14	3	2.1 ± 0.6 ^bcd^	0.2 ± 0.1 ^a^	56.7 ± 12.0 ^abc^	449 ± 110 ^ab^	NA	-
AA	Pisang Tongat	13	3	0.4 ± 0.1 ^d^	0.2 ± 0.1 ^a^	61.7 ± 7.3 ^ab^	527 ± 70.4 ^ab^	3	0
AA	Tapo	9	3	1.4 ± 1.1 ^cd^	0.2 ± 0.1 ^a^	100 ± 0.0 ^a^	869 ± 37.5 ^a^	NA	-
AA	Uwati	12	7	3.9 ± 0.9 ^abc^	0.1 ± 0.0 ^a^	33.3 ± 17.6 ^abc^	202 ± 117 ^bc^	24	0
AB	Chuoi Man	14	2	9.2 ± 1.6 ^a^	0.4 ± 0.1 ^a^	21.7 ± 11.7 ^bc^	187 ± 107 ^bc^	47	0
AB	Kunnan	15	18	2.5 ± 0.6 ^abc^	0.1 ± 0.0 ^a^	33.3 ± 6.7 ^abc^	80.6 ± 64.4 ^bc^	45	15.6
AB	Ney Poovan	15	36	2.5 ± 0.9 ^abc^	0.3 ± 0.1 ^a^	6.7 ± 6.7 ^c^	1.1 ± 1.1 ^c^	99	0
BB	Balbisiana Los Banos	8	NS	5.2 ± 1.9 ^ab^	0.1 ± 0.1 ^a^	0 ^c^	0 ^c^	7	0
Chisq				15.84	12.1	19.55	20.15	-	-
Df				8	8	8	8	-	-
*p*				0.04	0.15	0.01	0.009	-	-

NS = no symptoms; NA= not available (samples not available for testing due to plant death); BBTV = banana bunchy top virus; Data are means ± standard errors. Means with the same letter in a column are not significantly different (Bonferroni test, *p* = 0.05).

**Table 7:** Apterous and alate banana aphid abundance, banana bunchy top disease (BBTD) incidence, area under disease progress curve (AUDPC) and virus incidence on the triploid *Musa* genotypes after 36 months in the field.

Genomic Group	Genotypes	Total Plants Planted	Month to First Infection	Apterous Aphid Abundance	Alate Aphid Abundance	BBTD Incidence (%)	AUDPC	Samples Tested for BBTV	BBTV Incidence (%)
AAA	Gros Michel	10	21	5.4 ± 2.5 ^bcde^	0.22 ± 0.05 ^abc^	8.3 ± 8.3 ^a^	38.8 ± 38.8 ^ab^	23	4.2
AAA	Khai Thong Ruang	15	6	3.9 ± 0.9 ^bcde^	0.19 ± 0.05 ^bc^	40.0 ± 11.5 ^a^	261.8 ± 84.8 ^ab^	76	2.6
AAA	Williams	13	3	2.4 ± 0.5 ^de^	0.10 ± 0.03 ^c^	60.0 ± 10.0 ^a^	278.2 ± 108.0 ^ab^	6	16.7
AAA	Yagambi Km5	7	11	2.4 ± 1.3 ^de^	0.05 ± 0.03 ^c^	55.0 ± 5.0 ^a^	498.5 ± 51.8 ^a^	12	0
AAB	Batard	15	6	29.2 ± 6.7 ^a^	0.44 ± 0.05 ^ab^	20.0 ± 0.0 ^a^	98.5 ± 51.3 ^ab^	36	0
AAB	Ebang	15	16	21.4 ± 8.6 ^abc^	0.45 ± 0.12 ^ab^	40.0 ± 11.5 ^a^	186.6 ± 42.5 ^ab^	21	0
AAB	Elat	14	4	20.7 ± 3.8 ^ab^	0.37 ± 0.12 ^abc^	26.7 ± 17.6 ^a^	83.6 ± 76.4 ^ab^	32	0
AAB	Essong	15	4	23.5 ± 9.6 ^abcd^	0.55 ± 0.11 ^a^	33.3 ± 17.6 ^a^	253.1 ± 127.1 ^ab^	15	0
AAB	FHIA 25	15	4	2.0 ± 0.6 ^e^	0.11 ± 0.04 ^c^	66.7 ±17.6 ^a^	497.1 ± 128.7 ^a^	10	0
AAB	PITA 21	14	NS	19.9 ± 8.0 ^abcd^	0.34 ± 0.07 ^abc^	0 ^a^	0 ^b^	30	0
AAB	PITA 23	15	3	5.8 ± 1.5 ^abcde^	0.31 ± 0.09 ^abc^	53.3 ± 24.0 ^a^	348.4 ± 244.6 ^ab^	22	4.8
AAB	PITA 24	15	10	2.4 ± 0.8 ^e^	0.15 ± 0.04 bc	53.3 ± 17.6 ^a^	183.0 ± 89.9 ^ab^	23	4.2
AAB	PITA 27	12	7	9.5 ± 1.3 ^abcde^	0.27 ± 0.06 ^abc^	50.0 ± 14.4 ^a^	332.6 ± 69.4 ^ab^	16	0
AAB	Waema	15	2	9.3 ± 2.6 ^abcde^	0.28 ± 0.08 ^abc^	40.0 ± 11.5 ^a^	287.9 ± 53.7 ^ab^	44	0
ABB	Daru	15	4	13.9 ± 1.3 ^abcd^	0.32 ± 0.07 ^abc^	13.3 ± 6.7 ^a^	93.8 ± 56.3 ^ab^	48	4.2
ABB	Fougamou	11	36	3.3 ± 1.5 ^cde^	0.22 ± 0.06 ^abc^	8.3 ± 8.3 ^a^	19.2 ± 19.2 ^ab^	47	0
ABB	Pisang Awak	13	28	6.6 ± 1.7 ^abcde^	0.23 ± 0.06 ^abc^	6.7 ± 6.7 ^a^	1.1 ± 1.1 ^ab^	81	4.8
ABB	Balonkawe	10	NS	10.9 ± 3.6 ^abcde^	0.5 ± 0.2 ^abc^	0 ^a^	0 ^a^	9	0
BBB	Lep Chang Kut	7	15	6.2 ± 2.5 ^abcde^	0.01 ±0.01 ^c^	12.5 ± 12.5 ^a^	106.1 ± 106.1 ^ab^	3	0
Chisq				40.47	39.95	32.22	34.92	-	-
Df			-	18	18	18	18	-	-
*p*			-	0.002	0.002	0.02	0.01	-	-

NS = no symptoms; BBTV = banana bunchy top virus; Data are means ± standard errors. Means with the same letter in a column are not significantly different (Bonferroni test, *p* = 0.05).

**Table 8:** Apterous and alate banana aphid abundance, BBTD incidence, area under disease progress curve (AUDPC) and virus incidence on the tetraploid *Musa* genotypes after 36 months in the field.

Genomic Group	Genotypes	Total Plants Planted	Month to First Infection	Apterous Aphid Abundance	Alate Aphid Abundance	BBTD Incidence	AUDPC	Total Samples Tested for BBTV	BBTV Incidence (%)
AAAA	BITA2	9	14	4.9 ± 1.1 ^bc^	0.3 ± 0. 1 ^a^	55.6 ± 29.4 ^a^	317.6 ± 191.8 ^a^	6	0
AAAA	Buccaneer	14	3	7.2 ± 1.5 ^abc^	0.1 ± 0.0 ^a^	35.0 ± 5.0 ^a^	228.0 ± 46.5 ^a^	51	0
AAAA	FHI A23	15	4	6.5 ± 2.5 ^abc^	0.3 ± 0.1 ^a^	33.3 ± 6.7 ^a^	217.3 ± 51.0 ^a^	25	0
AAAA	SH 3436-6	13	4	3.7 ± 0.7 ^c^	0.3 ± 0.1 ^a^	48.3 ± 15.9 ^a^	338.1 ± 136 ^a^	15	17.8
AAAA	SH 3436-9	14	3	5.6 ± 2.0 ^bc^	0.2 ± 0.1 ^a^	50.0 ± 5.8 ^a^	335.6 ± 73.7 ^a^	11	8.3
AAAA	T6	14	3	3.0 ± 1.0 ^c^	0.1 ± 0.1 ^a^	33.3 ± 24.0 ^a^	197 ± 144 ^a^	9	0
AAAB	BITA 8	12	7	6.6 ± 1.3 ^abc^	0.4 ± 0.1 ^a^	25.0 ± 14.4 ^a^	169 ± 87.0 ^a^	17	0
AAAB	CRBP 37	15	26	1.97 ± 0.6 ^c^	0.1 ± 0.1 ^a^	6.7 ± 6.7 ^a^	23.1 ± 23.1 ^a^	19	0
AAAB	CRBP 39	15	11	24.0 ± 5.5 ^a^	0.3 ± 0.1 ^a^	33.3 ± 6.7 ^a^	168 ± 75.7 ^a^	14	6.7
AAAB	CRBP 535	14	28	21.0 ± 4.5 ^a^	0.5 ± 0.1 ^a^	8.3 ± 8.3 ^a^	28.9 ± 28.9 ^a^	25	0
AAAB	CRBP 568	15	18	9.2 ± 2.0 ^abc^	0.2 ± 0.1 ^a^	6.7 ± 6.7 ^a^	37.8 ± 37.8 ^a^	24	0
AAAB	CRBP 838	14	13	17.1 ± 4.1 ^ab^	0.3 ± 0.1 ^a^	25.0 ± 25.0 ^a^	146 ± 146 ^a^	35	2.6
AAAB	CRBP 969	15	4	24.9 ± 6.5 ^abc^	0.3 ± 0.1 ^a^	33.3 ± 17.6 ^a^	237 ± 119 ^a^	21	4.8
AAAB	FHIA 21	15	4	15.0 ± 3.2 ^abc^	0.4 ± 0.1 ^a^	53.3 ± 6.7 ^a^	323 ± 38.5 ^a^	9	0
AAAB	IRFA 908	15	4	7.9 ± 2.4 ^abc^	0.7 ± 0.4 ^a^	66.7 ± 13.3 ^a^	308 ± 84.9 ^a^	30	0
AABB	FHIA 03	7	3	14.5 ± 3.2 ^abc^	0.6 ± 0.1 ^a^	62.5 ± 37.5 ^a^	303 ± 46.8 ^a^	7	0
Df			-	16	16	16	16	-	-
*p*				0.003	0.18	0.14	0.16	-	-

BBTV = banana bunchy top virus; Data are means ± standard errors. Means with the same letter in a column are not significantly different (Bonferroni test, *p* = 0.05).

**Table 9:** Apterous and alate banana aphid abundance (mean ± SE) on the 12 *Musa* genotypes sorted from ploidy groups based on highest and lowest aphid abundance after 36 months in the field.

Genomic Groups	Genotypes	Apterous Aphids	Alate Aphids
Genotypes with high aphid densities			
AAB	Batard	29.2 ± 6.7 ^a^	0.4 ± 0.0 ^a^
AAAB	CRBP 969	24.9 ± 10.8 ^ab^	0.3 ± 0.1 ^ab^
AAAB	CRBP 39	24.0 ± 4.2 ^ab^	0.3 ± 0.1 ^ab^
AAB	Essong	23.5 ± 9.6 ^ab^	0.5 ± 0.0 ^ab^
AB	Chuoi Man	9.2 ± 2.2 ^abc^	0.4 ± 0.1 ^ab^
BB	Balbisiana Los Banos	5.2 ± 3.0 ^abcd^	0.1 ±0.0 ^b^
Genotypes with low aphid densities			
AAAA	T6	3.0 ± 1.0 ^bcd^	0.1 ± 0.1 ^ab^
AAA	Yagambi Km5	2.4 ± 1.4 ^bcd^	0.0 ± 0.0 ^b^
AAB	FHIA 25	2.0 ± 0.3 ^cd^	0.1 ± 0.0 ^b^
AAAB	CRBP 37	2.0 ± 0.8 ^cd^	0.1 ± 0.1 ^b^
AA	Tapo	1.2 ± 0.9 ^cd^	0.1 ± 0.1 ^b^
AA	Pisang Tongat	0.4 ± 0.2 ^d^	0.2 ± 0.1 ^ab^
Chisq		27.7	23.5
Df		11	11
*p*		0.004	0.02

Data are means ± standard errors. Means with the same letter in a column are not significantly different (Bonferroni test, *p* = 0.05).

**Table 10:** Banana bunchy top disease (BBTD) incidence and area under disease progress curve (AUDPC) (mean ± SE) of the 12 *Musa* genotypes sorted from three ploidy groups after 36 months in the field.

Genomic Group	Genotypes	Total Plants Planted	BBTD Incidence	AUDPC
AA	Tapo	9	100 ± 0.0 ^a^	869 ± 37.5 ^a^
AAB	FHIA 25	15	66.7 ± 17.6 ^a^	497 ± 128.7 ^ab^
AAAB	IRFA 908	15	66.7 ± 13.3 ^a^	308 ± 84.9 ^bc^
AABB	FHIA 03	7	62.5 ± 37.5 ^ab^	303 ± 46.8 ^abc^
AA	Pisang Tongat	13	61.7 ± 7.3 ^ab^	527 ± 70.4 ^ab^
AAA	Williams	13	60.0 ± 10.0 ^ab^	278 ± 108 ^bc^
AAAB	CRBP 568	15	6.7 ± 6.7 ^bc^	37.8 ± 37.8 ^cd^
AAAB	CRBP 37	15	6.7 ± 6.7 ^bc^	23.1 ± 23.1 ^cd^
AA	Calcutta 4	9	0 ^bc^	0 ^d^
BB	Balbisiana Los Banos	8	0 ^bc^	0 ^d^
ABB	Bolankawe	10	0 ^bc^	0 ^d^
AAB	PITA 21	14	0 ^bc^	0 ^d^
Chisq			26.9	28.5
Df			11	11
*p*			0.005	0.003

Data are means ± standard errors. Means with the same letter in a column are not significantly different (Bonferroni test, *p* = 0.05).

## Data Availability

Datasets generated during the study is available online: https://doi.org/10.25502/8107-0255/d (accessed on 28 March 2022), and the data summary presented in Supplementary Table S1.

## Supplementary Materials

The following supporting information can be downloaded at: https://www.mdpi.com/article/10.3390/plants11091206/s1, Table S1: Summary of *Musa* genotype response to banana aphid (*Pentalonia nigronervosa*) and banana bunchy top disease (BBTD) under screenhouse and field conditions.
